# Cost-benefit analysis of the integrated pharmaceutical supply chain information service after the establishment of the Korean Pharmaceutical Information Service

**DOI:** 10.3389/fphar.2022.925287

**Published:** 2022-10-26

**Authors:** Myojeong Kim, GeunWoo Lee, Yungi Hwang, Tae Hyun Kim, Dong-Sook Kim

**Affiliations:** ^1^ Department of Research, Health Insurance Review and Assessment Service, Wonju, South Korea; ^2^ College of Medicine, Yonsei University, Seoul Korea, South Korea

**Keywords:** cost-benefit analysis, pharmaceutical supply chain, pharmaceutical information service, Korea, benefit

## Abstract

**Background:** The Korean Pharmaceutical Information Service (KPIS) was established in October 2007 to increase the transparency of the pharmaceutical supply chain by integrating relevant information. This study aimed to describe the KPIS program and perform a cost-benefit analysis of the KPIS.

**Methods:** We conducted a cost-benefit analysis based on cost savings in terms of National Health Insurance (NHI). The outcome measures were the net financial benefit and benefit-cost ratio over the 12 years since the establishment of the KPIS. The cost estimate included the costs of labor and business operations, the development of an information entry system, and office maintenance. Financial benefits were defined as savings resulting from the implementation of the program based on KPIS data. Social benefits were defined as the prevention of recalled medicines from entering the supply chain and the decrease in inventory and disposal.

**Results:** The KPIS clearly resulted in a net financial benefit, saving 37.2 million USD, which was 2.6 times higher than the cost of implementation. While the benefit-cost ratio was less than one during the first period, it exceeded 3.4 during the second period. After calculating and integrating social benefits, the net benefit increased to 571.6 million USD, and the benefit-cost ratio was 24.8. A sensitivity analysis of the annual benefit showed that the net benefit varied from a low of −1.5 million USD to a high of 24.7 million USD according to the program implementation year.

**Conclusion:** The establishment of the KPIS and a system for collecting information on the pharmaceutical supply chain showed meaningful financial and social benefits when compared to the input cost. Since no other countries have an integrated pharmaceutical information system that incorporates all information from production to administration, the example of the KPIS can provide a precedent for other countries.

## Introduction

The growing role of medicines in healthcare systems globally is driven both by emerging innovative medicines and the expansion of access due to universal health coverage (Aitken, 2016). Pharmaceutical products, which are more commonly known as medicines or drugs, play a critical role in treating patients, making it necessary for each country to have a well-managed production and supply system (WHO, 1988). Therefore, numerous studies have aimed to identify effective strategies for optimizing these systems (Shah, 2004). The pharmaceutical value chain encompasses all organizational and operational activities needed to manufacture, distribute, and prescribe or dispense medicines to the end-user, beginning with development (Mendoza, 2021). Although the components of the value chain can differ between markets depending on the medicine type, distribution channel, reimbursement regulations, and region, the key stakeholders in the drug supply chain are almost always pharmaceutical companies (drug manufacturers), wholesale distributors, hospitals, pharmacies, third-party payers, and patients (Mendoza, 2021).

Since the pharmaceutical supply chain process has a crucial impact on medication quality and the final outcomes for patients, a recent innovative trend in the pharmaceutical sector has been the integrated management of medicines from production to distribution (HIRA et al., 2021). Introducing technology such as radiofrequency identification (RFID) to the pharmaceutical supply chain can guarantee transparency in the flow of drugs in terms of traceability, thereby improving communication, reducing counterfeiting, and enabling drug quality monitoring in pharmaceutical supply chains (Catarinucci et al., 2012). Counterfeit and potentially harmful drugs are a growing problem worldwide, costing the pharmaceutical industry approximately 10% of its total revenue and contributing to numerous patient deaths (Mackey & Nayyar, 2017). Many countries have attempted to prevent counterfeit drugs from entering the pharmaceutical supply chain. On 27 November 2013, US President Barack Obama signed into law Title II of the Drug Quality and Security Act, now known as the Drug Supply Chain Security Act (DSCSA). The DSCSA requires the pharmaceutical supply chain to implement medication tracking and tracing; serialization, verification, and detection of suspicious products; and strict guidelines for wholesaler licensing and reporting (Brechtelsbauer et al., 2016) through the creation of programs such as the California E-Pedigree drug tracing program (Barlas, 2011; Mackey & Liang, 2011).

In South Korea, the government established the Korean Pharmaceutical Information Service (KPIS) in October 2007 under the jurisdiction of the Health Insurance Review and Assessment Service (HIRA) in order to implement an information management system that effectively integrates and tracks pharmaceutical information, codes, and supply chain data. In 2020, there were 435 manufacturers and importers and 3,108 wholesalers of drugs in Korea (HIRA, Annual). The HIRA determines the maximum reimbursement price for medicines through the National Health Insurance (NHI) and pays for the actual transaction cost of medicines in hospitals and pharmacies (Roughead et al., 2018). Before the KPIS was established, information about the importing, supply, and dispensing of pharmaceuticals were handled by several different agencies including the Ministry of Food and Drug Safety, the Ministry of Health and Welfare, and the HIRA. Moreover, due to inconsistent approval codes, supply codes, and NHI drug codes, a system of codes was needed to easily identify manufacturers and drug categories. Accordingly, the government revised the Pharmaceutical Affairs Law in October 2007 and (Barchetti et al., 2010) established the KPIS.

**FIGURE 1:**
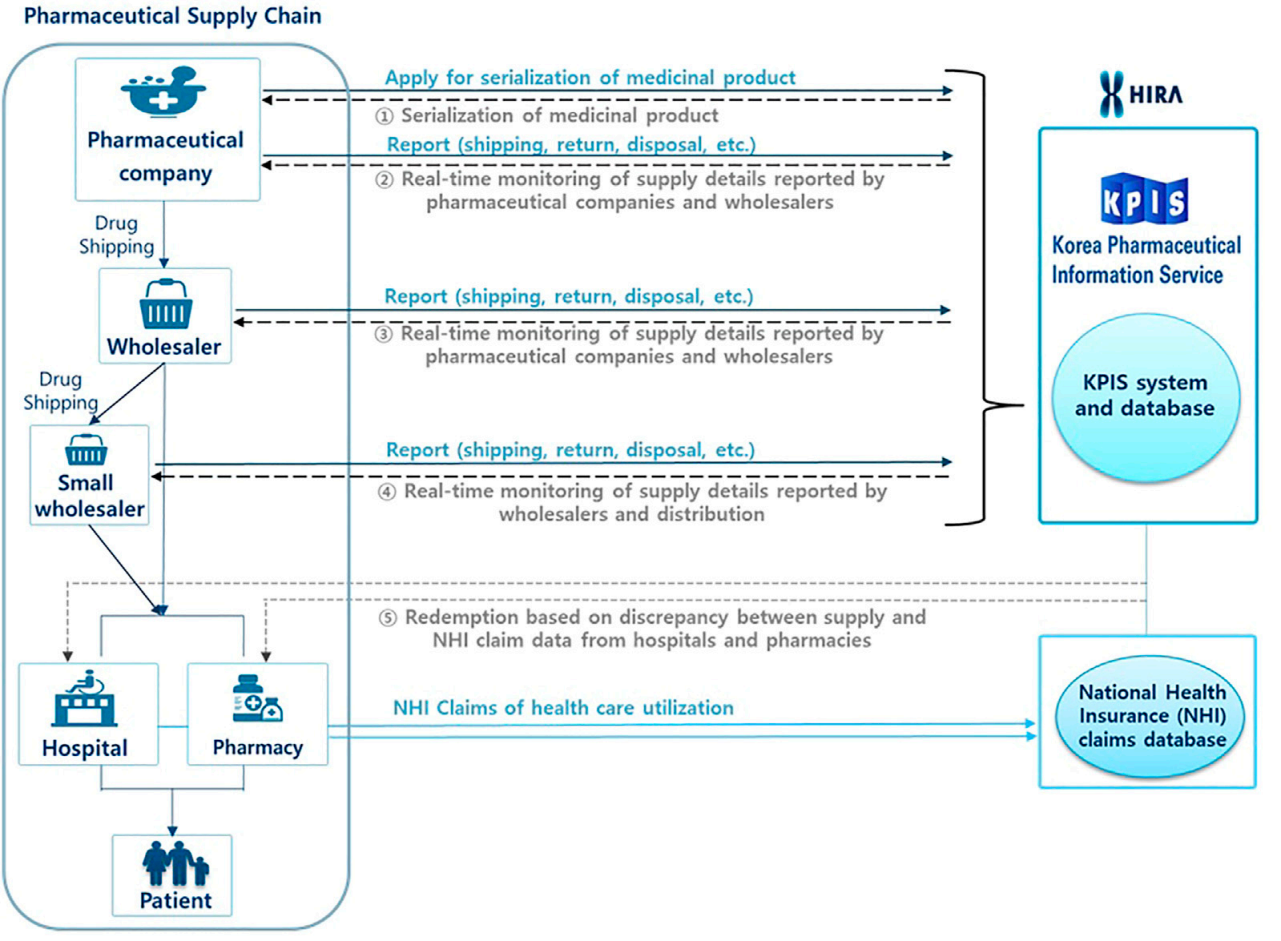
Schematic diagram of the functions of the Korean Pharmaceutical Information Service (KPIS).

Figure 1 shows the pharmaceutical supply chain covering from manufacturing and distribution to consumption to patients and the role of KPIS. The KPIS has conducted several projects such as 1) the serialization of individual products using 13-digit codes, 2) the real-time monitoring of supply details reported by pharmaceutical companies and wholesalers on a daily basis, including information on shipping, returns, and disposal, 3) overseeing the management of barcodes and RFID, 4) inspections to compare NHI claims data from hospitals or pharmacies against KPIS data from wholesalers, and 5) inspections to determine the actual transaction costs of medicines by comparing NHI claims data and KPIS data (Figure 1). If there is a large discrepancy between the wholesalers’ data on their supply and the claims data from hospitals and pharmacies, the KPIS initiates an investigation. In addition, if a pharmacy claims the maximum price of a medication rather than the average purchasing price, then the KPIS asks the HIRA to determine the actual amount.

While the objective of the KPIS is to enhance safe medication use and to promote transparency within the pharmaceutical supply chain, there have been no studies to evaluate its achievements during the 12 years for which it has existed. Therefore, this study conducted a cost-benefit analysis of the KPIS since its establishment in terms of efficiency and medicine safety.

## Meterial and methods

### Study design and outcome measures

We conducted a cost-benefit analysis to compare uniform measurements using monetary values to evaluate the effect of projects carried out by the KPIS. The model was framed from the perspective of the NHI.

The primary outcome measures were the net financial benefit and benefit-cost ratio over the 12 years since the establishment of the KPIS. Data on costs and benefits were obtained using KPIS data, NHI claims data, and other published studies.

The formula used in this study is shown below (1). The net present value is the difference between benefits and costs, and a difference of greater than 0 indicates that there were some cost savings. The net present value was obtained by calculating the net benefit (benefits minus costs) according to the inflation rate of the corresponding year. The benefit-cost ratio is also shown below (2). To derive this formula, the benefits and costs generated during the period of each project were converted into the present value in 2018 according to the inflation rate of the corresponding year. Then, the sum of the benefits for each project was divided by the sum of each project’s costs.

$$\text{Net}\;\text{present}\;\text{value}=\overset{2018}{\underset{t=2007}{\sum }}\left\{B_{t}\times \left(\frac{cpi_{2018}}{cpi_{t}}\right)\right\}-\overset{2018}{\underset{t=2007}{\sum }}\left\{C_{t}\times \left(\frac{cpi_{2018}}{cpi_{t}}\right)\right\}$$ (1)

$$B/C=\frac{\overset{2018}{\underset{t=2007}{\sum }}\left\{B_{t}\times \left(\frac{cpi_{2018}}{cpi_{t}}\right)\right\}}{\overset{2018}{\underset{t=2007}{\sum }}\left\{C_{t}\times \left(\frac{cpi_{2018}}{cpi_{t}}\right)\right\}}$$ (2)

*B*_*t*_ and *C*_*t*_ represent the benefits and costs generated in each selected yearn

*Cpi*_*t*_ represents the consumer price index at the time (t).

In addition, we divided the study period into two segments (2007-2012 and 2013-2018). The process for investigating fraud claims using KPIS data and NHI claims data was revised in 2014, which could have had a substantial effect on the benefit of the KPIS. Therefore, we analyzed data from 2007 to 2018 (the entire period), 2007 to 2012 (the first period), and 2013 to 2018 (the second period).

### Cost estimate

The cost estimate was defined as expenses related to the operation of the KPIS and was mainly based on financial accounting statements for each fiscal year. We estimated the cost across 3 areas: 1) labor and operation costs, 2) the development of the information entry system (including the cost of storage devices and software), and 3) building depreciation, real estate taxes, and office maintenance. We calculated both the nominal cost and the present value based on the consumer price index for 2018. The cost is presented in Table 1.

**TABLE 1:** Parameter values in the model.

Parameter (million USD)	Base case				
	Entire period	First period (2007-2012)<	Second period (2013-2018)	Annual value (range)	Source
**Cost**					
*Total cost*	24.0	8.8	15.2	2.0 (0.9–3.6)	
Labor and operation costs	14.1	3.7	10.5	1.2 (0.2–2.7)	Financial accounting
Information entry system hardware and software	9.0	4.8	4.2	0.7 (0.6–0.9)	Financial accounting
Office maintenance costs	0.9	0.3	0.6	0.1 (0.0–0.1)	Financial accounting
**Financial benefits**					
*Savings from program* (Comparison of discrepancies between wholesalers’ supply and NHI claims data from hospitals and pharmacies)	61.2	9.2	52.0	5.1 (0.9–3.6)	
Fee for providing KPIS information	8.1	3.0	4.7	0.7 (0.3–1.3)	Report
Providing KPIS information to the government (saving without survey)	0.9	0.2	0.7	0.1 (0.1–0.1)	Analysis
Detection of errors in reporting supply detail	20.5	0.0	20.5	1.7 (0–20.5)	Report
Inspection of claims of medicine price	12.2	5.6	6.6	1.0 (0.7–3.8)	Report
Inspection of recalled medicines distribution (1 year)	2.2	-	2.2	0.2 (0–2.2)	Report
Pharmaceutical consumption statistics (cost savings without survey)	1.3	0.0	1.2	0.1 (0–0.3)	Report
Prevention of rebates to doctors by drug companies	16.0	0.0	16.0	1.3 (0–3.2)	Report
**Social benefits**					
Prevention of recalled medicines use	171.4				Analysis
Improvement of the efficiency of the supply chain	361.8				Alshemari et al. (2020)

KPIS, korean pharmaceutical information service; NHI, National Health Insurance; Note: Measurements are based on the year 2018, reflecting the consumer price index.

The labor, business operation, and computer server costs were estimated based on separate KPIS financial accounting reports for each fiscal year. However, the cost estimates for building depreciation, real estate taxes, and office maintenance were included in the general accounting records of the HIRA, and they were determined by calculating the proportional cost of the KPIS (1.4%) based on the general accounting records of the HIRA.

## Benefit estimate

The benefit estimate was defined in terms of NHI financial benefits and social benefits. The financial benefits were measured as the savings resulting from the implementation of the program and the commission fee for providing KPIS information. The financial savings were derived based on 1) a comparison of the large discrepancy between data from wholesalers and claims data from hospitals and pharmacies, 2) an investigation of the actual transaction cost of medicine using KPIS data, 3) savings from decreased medicine prices resulting from the ability to identify rebates offered to doctors by pharmaceuticals companies. Social benefits were defined as the prevention of recalled medicines from entering the supply chain and the reduction of inventory and disposal.

Data were obtained from a KPIS report that analyzed both NHI claims data and KPIS data from 2007 to 2018. The KPIS created a dataset based on data from 35,054 medical institutions (general hospitals, hospitals, and clinics), 17,905 dental hospitals and clinics, 3,478 public health agencies, and 22,082 pharmacies across the entire population of South Korea, at 50.8 million people. We calculated the expenses in order to quantify the financial benefits and savings.

Social benefits were defined based on the potential costs reported in previous studies. We conducted a literature review of articles published between 2005 and 2019 in Korean or English using Google Scholar, IEEE Xplore, and Medline. We also reviewed texts published by the government or research centers, or works published in newspapers.

Finally, we defined financial benefits as savings from the implementation of the program and social benefits as the improvement of patient safety and the increase in transparency of the distribution chain. First, since monitoring reports of supply details and providing information about recalled medicines could prevent recalled medicines from entering the supply chain, thereby tracking and integrating information on the pharmaceutical chain could enhance patient safety, we analyzed the drug cost of recalled medicines in the previous year. Second, we used the value on the improvement of the efficiency of the supply chain in the literature review.

### Base analysis and subgroup analysis

We analyzed the net financial benefit and benefit-cost ratio for the entire period, the first period, and the second period. In addition, we compared the results for financial benefits alone with the results for financial benefits and social benefits combined. Sensitivity analysis was performed using all possible combinations of savings resulting from the program’s implementation according to the annual range at the end of each year.

## Results

### Cost and benefit of the implementation of KPIS programs

The overall values for costs and benefits are shown in Table 1, and the yearly values are shown in Table 2.

**TABLE 2:** Twelve-year costs and savings from the implementation of KPIS program (million USD).

Year	2007	2008	2009	2010	2011	2012	2013	2014	2015	2016	2017	2018
**Cost**												
Labor and operation costs	0.0	0.8	1.0	1.0	0.3	0.5	0.2	0.8	1.7	2.4	2.7	2.6
Information entry system hardware and software	0.9	0.8	0.9	0.6	0.8	0.8	0.6	0.6	0.7	0.8	0.8	0.8
Office maintenance costs	0.0	0.0	0.1	0.1	0.0	0.1	0.1	0.1	0.1	0.1	0.1	0.1
**Financial benefits**												
Fee for providing KPIS information		0.3	0.5	0.6	0.9	1.0	1.3	1.1	0.8	0.7	0.6	0.3
Providing KPIS information to the government (saving without survey)					0.1	0.1	0.1	0.1	0.1	0.1	0.1	0.1
Detection of errors in reporting supply detail								20.5			0.0	0.0
Inspection of claims of medicine price					1.8	3.8	2.5	0.8	0.7	0.7	0.9	0.9
Inspection of recalled medicines distribution (1 year)												2.2
Pharmaceutical consumption statistics (cost savings without survey)								0.2	0.3	0.3	0.2	0.3
Prevention of rebates to doctors by drug companies							3.2	3.4	2.9	2.3	2.2	2.1
**Social benefits**												
Prevention of recalled medicines use*											80.7	90.7
Improvement of the efficiency of the supply chain											120	240

KPIS, korean pharmaceutical information service; NHI, National Health Insurance; Note: The cost of preventing recalled medicines from entering the supply chain in 2019 was 298.2 million USD.

The labor and operation cost totaled 14.1 million USD, while the cost to develop the information entry system (including storage devices and software cost) was 9.0 million USD. The cost estimate for building depreciation, real estate taxes, and office maintenance was 0.9 million USD.

Savings from the implementation of the program totaled 61.2 million USD. The commission fee for providing KPIS data to companies was 8.1 million USD, and savings from the reduction of the large discrepancy between data from wholesalers and NHI claims from hospitals or pharmacies was 20.5 million USD. The decrease in expenditures due to lower drug prices based on actual transaction costs was 12.2 million USD, and savings from the decreased cost of medicines due to the ability to identify rebates offered to doctors by pharmaceutical companies totaled 16 million USD.

## Net benefit and benefit-cost ratio

We calculated the net present value as of 2018 using the financial savings from the KPIS program (Table 3). Over the entire period, the KPIS had a substantial financial net benefit of 37.2 million USD. At the beginning of the program, the initial costs were paid; therefore, the savings were not yet apparent. Thus, the first period analyzed (2007-2012) did not correspond to substantial revenue from the various projects undertaken by the KPIS, and the financial benefit was mostly seen during the second period (2013-2018). Throughout the entire period, the benefit was 2.6 times higher than the cost. The benefit-cost ratio was less than 1 in the first period, and it exceeded 3.4 during the second period.

**TABLE 3:** Results of the cost-benefit analysis of the base case and scenarios.

	Net benefit (million USD)		Benefit-cost ratio	
	Cumulative value	Annual value	Cumulative value	Annual value
**Financial benefits**				
Entire period	37.2	3.1 (−1.5–24.7)	2.6	3.2 (0–18.2)
First period (2007-2012)	0.4	0.0 (−1.5–3.6)	1.0	1.1 (0–3.7)
Second period (2013-2018)	36.8	6.2 (0.4–24.7)	3.4	5.4 (1.2–18.2)
**Financial and social benefits** (improvement of the efficiency and patient safety)				
Entire period	571.6	55.2 (−1.5–333.2)	24.8	15.8 (0–96.6)
First period (2007-2012)	0.4	0.0 (−1.5–3.6)	1.0	1.1 (0–3.7)
Second period (2013-2018)	571.2	95.2 (0.8–333.2)	38.5	30.5 (1.2–96.6)

**FIGURE 2:**
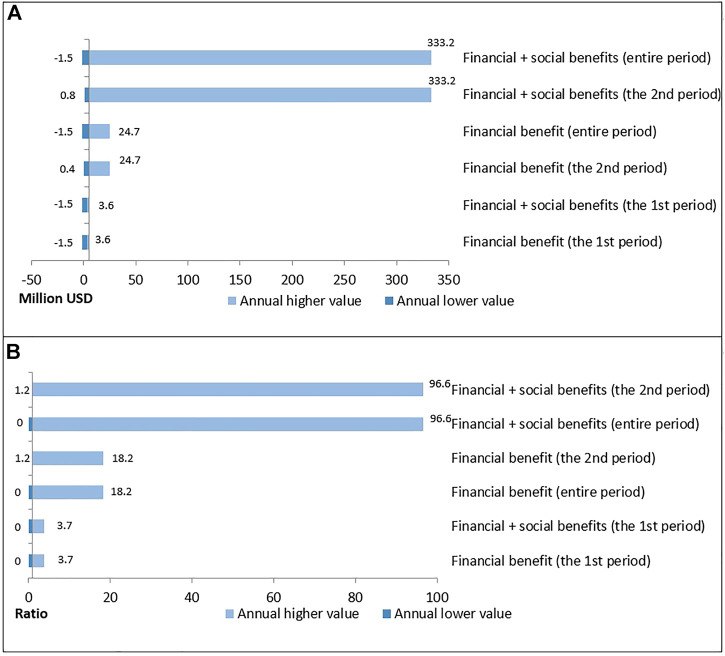
A tornado diagram of the sensitivity analysis.

When the social benefits and financial benefits were combined, the net benefit increased to 441.7 million USD, and the benefit-cost ratio was 19.4 (Figure 2).

## Discussion

Since the integration of information on the pharmaceutical supply chain prompted by the creation of the KPIS in October 2007, the KPIS has been able to track the serial numbers of medicines from the production stage to distribution as a result of the serialization of all medicines. The KPIS has conducted several projects such as monitoring supply details reported by pharmaceutical companies and wholesalers on a daily basis, including information on shipping, returns, and disposal; managing barcodes and RFIDs; and calculating the amount of supply and actual transaction cost of medicines using NHI claims data and KPIS data.

Previous studies have suggested that a drug traceability system incorporated into the pharmaceutical supply chain can create value (Silva & Mattos, 2019), and the integration of supply chain information should be viewed as an effective risk management tool for mitigating uncertainty and risk in the supply chain (Wang & Jie, 2020). Another study suggested that implementing an RFID model for tracking drugs at the item level in the pharmaceutical supply chain might have the potential to reduce the scope of the counterfeit drug problem (Coutstasse et al., 2010). One study also suggested that a circular pharmaceutical supply chain might reduce medicine waste (Alshemari et al., 2020). Risks in the pharmaceutical supply chain include product discontinuity, product shortages, poor performance, patient safety/dispensing errors, and technological errors (resulting in stock shortages at pharmacies). Strategies for optimizing the pharmaceutical supply chain can be achieved through the integration of information (Shah, 2004).

According to this study, over the 12 years since the establishment of the KPIS, despite the initial costs, the KPIS and the integration of information on the pharmaceutical supply chain have shown clear benefits from the various programs administered by the KPIS and generated social benefits including the prevention recalled medicines from reaching the market and improving the efficiency of the supply chain. Our study showed that, although the initial costs of the information entry system were high, including the costs of a super-computer server and software, the benefits of the system and program are also high. Although the value of integrated information on the pharmaceutical supply chain may be high, it is challenging to create a system that incorporates information on authorization, supply, distribution, and reimbursement across various medical institutions, pharmacies, and wholesalers. Nonetheless, the implementation of such a system can be expected to provide crucial benefits in terms of transparency of the pharmaceutical supply chain as well as patient safety. Especially transparency of the pharmaceutical supply chain might be strengthened from the program such as reporting drug shipping, return, and disposal, *etc.* By pharmaceutical companies or wholesalers and integrating these data, thereby cost savings can occur. The inspections to compare the medicine use in the NHI claims data from hospitals or pharmacies against the supply details in the KPIS data from wholesalers could detect errors or fraud of claims. Also, by integrating information on the pharmaceutical supply chain, tracking the potentially harmful drugs use such as recalled medicines could improve patient safety.

Since no other countries have comparable systems or institutions to the KPIS that integrate information on the pharmaceutical supply chain from the manufacturing stage to administration, it is difficult to compare the effect of this program for enhancing the transparency of the pharmaceutical supply chain to other systems. Several studies found that risks in the pharmaceutical supply chain were internal risks that could be managed using mitigation strategies (Jaberidoost et al., 2013; Wang & Jie, 2020), and efforts should be made to prevent pharmaceutical counterfeiting from entering the supply chain using RFID technology. However, no studies have yet evaluated the outcomes of establishing an integrated information system related to pharmaceutical supply chain management and logistics.

To the best of our knowledge, this is the first study to describe the model for integrating and managing information on the pharmaceutical supply chain in Korea and conduct a cost-benefit analysis of the KPIS to gather information and implement programs using these data. Thus, the results provide meaningful evidence for the establishment of pharmaceutical supply chain information management systems. We analyzed the costs and savings related to the prevention of recalled medicines and hazardous drugs containing carcinogenic substances from entering the supply chain by examining pharmaceutical expenditures from previous years using KPIS data. In addition, we attempted to consider social benefits based on improvements in the efficiency of the supply chain.

Second, this system such as KPIS is expected to be a reference for other countries in their efforts to integrate and manage information on the pharmaceutical supply chain. Moreover, as patient-oriented care increases in importance and access to pharmaceutical information expand to become more consumer-oriented and easier, meaning that the system integrating the pharmaceutical supply chain information will play a critical role as a provider of information on the pharmaceutical supply chain.

Nevertheless, our study has limitations. We limited the financial and social benefits to empirically measured values and only used an estimated value to measure improvement in the efficiency of the supply chain. Second, the time and costs related to entering information into the entry system by pharmaceutical companies and wholesalers were not included when estimating costs since this study focused on the costs paid through NHI and the related savings. Second, the time and costs related to entering information into the entry system by some players (pharmaceutical companies, wholesalers) in the PSC were not included when estimating costs since the model in this study was framed from the perspective of the NHI, and so we focused on the costs paid through NHI and the related savings. Also, in terms of benefits, we did not include the benefit of the pharmaceutical companies and wholesalers using the KPIS data.

In conclusion, the establishment of the KPIS and a system for collecting information on the pharmaceutical supply chain in Korea showed improved benefits compared to the cost of implementation. Further strategies should be introduced to increase the efficiency of the pharmaceutical supply chain and to promote patient safety by providing patients, physicians, and pharmacists with supply and distribution information.

## Data Availability

The original contributions presented in the study are included in the article/Supplementary Material, further inquiries can be directed to the corresponding author.
